# Supplementary material for: Is Fibersol-2 efficacious in reducing duration of watery diarrhea and stool output in children 1–3 years old? A randomized, parallel, double-blinded, placebo-controlled, two arm clinical trial
Source: PLoS One. 2023 Jan 27;18(1):e0280934. doi: 10.1371/journal.pone.0280934 (PMC9882758; doi:10.1371/journal.pone.0280934)
Supplement: S1 Appendix — (DOCX) [file pone.0280934.s002.docx]

**S1 Appendix**

**Data availability**

The data set contained personal information of the study participants. Our institutional review board will not have the provision to disclose any kind of information. Thus, our policy is not to make availability of the data set in the manuscript, the supplemental files, or a public repository. However, data related to this manuscript are available upon request and for researchers who meet the criteria for access to confidential data may contact with Ms. Armana Ahmed ([armana@icddrb.org](mailto:armana@icddrb.org)) to the research administration of icddr,b (<http://www.icddrb.org/>).

**Table S1. Resolution of diarrhea from 1^st^ to 21^st^ 8-hour period of time**

| **Variable of interest** | **Placebo (n=47)** | **Fibersol-2 (n=45)** | **p-value** |
| --- | --- | --- | --- |
| Resolution of diarrhea 1^st^ 8 hours | 3 (6) | 4 (9) | 0.952 |
| Resolution of diarrhea 2^nd^ 8 hours | 10 (21) | 6 (13) | 0.226 |
| Resolution of diarrhea 3^rd^ 8 hours | 11 (23) | 10 (22) | 0.717 |
| Resolution of diarrhea 4^th^ 8 hours | 3 (6) | 4 (9) | 0.952 |
| Resolution of diarrhea 5^th^ 8 hours | 5 (11) | 4 (9) | 0.945 |
| Resolution of diarrhea 6^th^ 8 hours | 6 (13) | 5 (11) | 0.793 |
| Resolution of diarrhea 7^th^ 8 hours | 1 (2) | 0 (0) | 1.000 |
| Resolution of diarrhea 8^th^ 8 hours | 1 (2) | 2 (4) | 0.578 |
| Resolution of diarrhea 9^th^ 8 hours | 3 (6) | 2 (4) | 0.641 |
| Resolution of diarrhea 10^th^ 8 hours | 1 (2) | 1 (2) | 0.493 |
| Resolution of diarrhea 11^th^ 8 hours | 1 (2) | 0 (0) | 0.982 |
| Resolution of diarrhea 12^th^ 8 hours | 1 (2) | 0 (0) | 0.982 |
| Resolution of diarrhea 13^th^ 8 hours | 0 (0) | 1 (2) | 0.983 |
| Resolution of diarrhea 14^th^ 8 hours | 0 (0) | 2 (4) | 0.225 |
| Resolution of diarrhea 15^th^ 8 hours | 1 (2) | 3 (7) | 0.969 |
| Resolution of diarrhea 16^th^ 8 hours | 0 (0) | 0 (0) | - |
| Resolution of diarrhea 17^th^ 8 hours | 0 (0) | 0 (0) | - |
| Resolution of diarrhea 18^th^ 8 hours | 0 (0) | 0 (0) | - |
| Resolution of diarrhea 19^th^ 8 hours | 0 (0) | 1 (2) | 0.983 |
| Resolution of diarrhea 20^th^ 8 hours | 0 (0) | 0 (0) | - |
| Resolution of diarrhea 21^st^ 8 hours | 0 (0) | 0 (0) | - |

Values are expressed as number of subjects as n; p-value was assessed by t-test or Chi-square test

**Table S2. Stool consistency in both the groups**

| **Variables** | **Placebo (n=47)** | | **Fibersol-2 (n=45)** | **p-value** |
| --- | --- | --- | --- | --- |
| Consistency of stool 1^st^ 8 hours | | | | |
| Solid | | 0 (0) | 0 (0) | - |
| Paste | | 0 (0) | 0 (0) | - |
| Loose | | 46 (98) | 43 (95) | 0.484 |
| Consistency of stool 2^nd^ 8 hours | | | | |
| Solid | | 0 (0) | 0 (0) | - |
| Paste | | 4 (8) | 3 (7) | 0.751 |
| Loose | | 40 (85) | 32 (71) | 0.751 |
| Consistency of stool 3^rd^ 8 hours | | | | |
| Solid | | 0 (0) | 0 (0) | - |
| Paste | | 8 (17) | 5 (11) | 0.539 |
| Loose | | 27 (57) | 30 (67) | 0.539 |
| Consistency of stool 4^th^ 8 hours | | | | |
| Solid | | 0 (0) | 1 (2) | 0.997 |
| Paste | | 20 (42) | 14 (31) | 0.335 |
| Loose | | 16 (34) | 19 (42) | 0.473 |
| Consistency of stool 5^th^ 8 hours | | | | |
| Solid | | 1 (2) | 2 (4) | 1.00 |
| Paste | | 13 (28) | 12 (27) | 1.00 |
| Loose | | 17 (36) | 17 (38) | 0.799 |
| Consistency of stool 6^th^ 8 hours | | | | |
| Solid | | 2 (4) | 1 (2) | 0.573 |
| Paste | | 15 (32) | 13 (29) | 0.976 |
| Loose | | 13 (27) | 11 (24) | 0.927 |
| Consistency of stool 7^th^ 8 hours | | | | |
| Solid | | 4 (8) | 5 (11) | 0.907 |
| Paste | | 16 (34) | 13 (29) | 0.417 |
| Loose | | 7 (15) | 11(24) | 0.499 |
| Consistency of stool 8^th^ 8 hours | | | | |
| Solid | | 4 (8) | 5 (11) | 0.959 |
| Paste | | 20 (42) | 14 (31) | 0.259 |
| Loose | | 8 (17) | 12 (27) | 0.369 |
| Consistency of stool 9^th^ 8 hours | | | | |
| Solid | | 4 (8) | 5 (11) | 0.77 |
| Paste | | 11 (23) | 9 (20) | 0.413 |
| Loose | | 6 (13) | 11 (24) | 0.439 |
| Consistency of stool 10^th^ 8 hours | | | | |
| Solid | | 9 (19) | 9 (20) | 0.847 |
| Paste | | 13 (28) | 13 (29) | 0.887 |
| Loose | | 6 (13) | 5 (11) | 0.946 |
| Consistency of stool 11^th^ 8 hours | | | | |
| Solid | | 5 (11) | 8 (18) | 0.682 |
| Paste | | 13 (28) | 13 (29) | 0.91 |
| Loose | | 8 (17) | 8 (18) | 0.97 |
| Consistency of stool 12^th^ 8 hours | | | | |
| Solid | | 9 (19) | 6 (13) | 0.566 |
| Paste | | 9 (19) | 11 (24) | 0.413 |
| Loose | | 7 (15) | 5 (11) | 0.988 |
| Consistency of stool 13^th^ 8 hours | | | | |
| Solid | | 11 (23) | 11 (24) | 0.866 |
| Paste | | 16 (34) | 11(24) | 0.244 |
| Loose | | 2 (4) | 8 (18) | 0.094 |
| Consistency of stool 14^th^ 8 hours | | | | |
| Solid | | 9 (19) | 5 (11) | 0.213 |
| Paste | | 11 (23) | 14 (31) | 0.906 |
| Loose | | 2 (4) | 6 (13) | 0.333 |
| Consistency of stool on 15^th^ 8 hours | | | | |
| Solid | | 10 (21) | 11 (24) | 0.615 |
| Paste | | 6 (13) | 12 (27) | 0.538 |
| Loose | | 1 (2) | 1 (2) | 0.628 |
| Consistency of stool 16^th^ 8 hours | | | | |
| Solid | | 20 (42) | 13 (29) | 0.217 |
| Paste | | 8 (17) | 11 (24) | 0.499 |
| Loose | | 0 () | 2 (4) | 0.454 |
| Consistency of stool 17^th^ 8 hours | | | | |
| Solid | | 18 (38) | 7 (15) | 0.349 |
| Paste | | 8 (17) | 7 (15) | 0.442 |
| Loose | | 1 (2) | 1 (2) | 0.746 |
| Consistency of stool 18^th^ 8 hours | | | | |
| Solid | | 12 (25) | 13 (29) | 0.618 |
| Paste | | 6 (13) | 8 (18) | 0.741 |
| Loose | | 0 (0) | 3 (7) | 0.341 |
| Consistency of stool 19^th^ 8 hours | | | | |
| Solid | | 24 (51) | 19 (42) | 0.745 |
| Paste | | 8 (17) | 7 (15) | 0.765 |
| Loose | | 0 (0) | 2 (4) | 0.414 |
| Consistency of stool 20^th^ 8 hours | | | | |
| Solid | | 12 (25) | 13 (29) | 0.851 |
| Paste | | 3 (6) | 2 (4) | 0.879 |
| Loose | | 0 (0) | 2 (4) | 0.522 |
| Consistency of stool 21^st^ 8 hours | | | | |
| Solid | | 16 (34) | 15 (33) | 0.166 |
| Paste | | 2 (4) | 5 (11) | 0.632 |
| Loose | | 0 (0) | 3 (7) | 0.323 |

Values are expressed as number of subjects as n; p-value was assessed by t-test or Chi-square test

**Table S3. Amount of stool output per person in both groups (gram)**

| **Variables (mean±SD)** | **Placebo (n=47)** | **Fibersol-2 (n=45)** | **p-value** |
| --- | --- | --- | --- |
| Amount of stool 1^st^ 8 hours | 145.07±113.39 | 191.05±176.62 | 0.553 |
| Amount of stool 2^nd^ 8 hours | 118.55±61.66 | 151.77±120.89 | 0.823 |
| Amount of stool 3^rd^ 8 hours | 127.67±84.50 | 112.20±78.21 | 0.279 |
| Amount of stool 4^th^ 8 hours | 100.36±94.89 | 134.71±131.55 | 0.613 |
| Amount of stool 5^th^ 8 hours | 104.10±69.45 | 105.78±69.61 | 0.740 |
| Amount of stool 6^th^ 8 hours | 76.50±57.26 | 96.50±55.06 | 0.070 |
| Amount of stool 7^th^ 8 hours | 78.59±39.47 | 109.34±111.34 | 1.000 |
| Amount of stool 8^th^ 8 hours | 83.22±75.65 | 83.74±65.78 | 0.778 |
| Amount of stool 9^th^ 8 hours | 78.86±52.01 | 94.52±76.41 | 0.543 |
| Amount of stool 10^th^ 8 hours | 69.89±26.56 | 87.48±91.64 | 0.685 |
| Amount of stool 11^th^ 8 hours | 99.77±61.90 | 82.55±52.46 | 0.214 |
| Amount of stool 12^th^ 8 hours | 80.28±65.68 | 88.86±68.42 | 0.454 |
| Amount of stool 13^th^ 8 hours | 68.48±35.92 | 84.33±82.03 | 0.658 |
| Amount of stool 14^th^ 8 hours | 58.55±32.56 | 106.08±67.61 | 0.003 |
| Amount of stool 15^th^ 8 hours | 62.71±46.13 | 92.88±92.97 | 0.353 |
| Amount of stool 16^th^ 8 hours | 58.37±33.67 | 67.62±35.08 | 0.304 |
| Amount of stool 17^th^ 8 hours | 78.30±76.57 | 89.80±41.84 | 0.060 |
| Amount of stool 18^th^ 8 hours | 55.33±25.30 | 76.88±57.29 | 0.145 |
| Amount of stool 19^th^ 8 hours | 65.56±39.58 | 75.64±51.47 | 0.293 |
| Amount of stool 20^th^ 8 hours | 52.07±26.71 | 60.41±26.26 | 0.352 |
| Amount of stool 21^st^ 8 hours | 58.61±46.74 | 67.65±30.67 | 0.061 |

Values are expressed as mean±SD; p-value was assessed by t-test or Chi-square test; SD, Standard deviation

**Table S4. Stool frequency per person in both groups**

| **Variables (mean±SD)** | **Placebo (n=47)** | **Fibersol-2 (n=45)** | **p-value** |
| --- | --- | --- | --- |
| Frequency of stool 1^st^ 8 hours | 4.46±3.45 | 4.72±3.45 | 0.293 |
| Frequency of stool 2^nd^ 8 hours | 3.70±2.22 | 3.86±2.45 | 0.352 |
| Frequency of stool 3^rd^ 8 hours | 3.71±3.07 | 2.83±2.40 | 0.061 |
| Frequency of stool 4^th^ 8 hours | 2.72±2.12 | 2.91±2.62 | 0.776 |
| Frequency of stool 5^th^ 8 hours | 3.23±3.19 | 2.29±2.10 | 0.077 |
| Frequency of stool 6^th^ 8 hours | 2.07±1.91 | 2.21±1.64 | 0.409 |
| Frequency of stool 7^th^ 8 hours | 2.04±1.56 | 2.79±2.51 | 0.497 |
| Frequency of stool 8^th^ 8 hours | 2.06±1.80 | 2.29±1.77 | 0.258 |
| Frequency of stool 9^th^ 8 hours | 1.81±1.37 | 2.56±2.10 | 0.191 |
| Frequency of stool 10^th^ 8 hours | 1.82±1.19 | 2.52±2.50 | 0.457 |
| Frequency of stool 11^th^ 8 hours | 2.46±2.60 | 1.90±1.50 | 0.640 |
| Frequency of stool 12^th^ 8 hours | 1.76±2.24 | 1.76±1.73 | 0.889 |
| Frequency of stool 13^th^ 8 hours | 1.14±0.35 | 1.70±1.69 | 0.035 |
| Frequency of stool 14^th^ 8 hours | 1.50±0.91 | 2.04±1.37 | 0.132 |
| Frequency of stool 15^th^ 8 hours | 1.12±0.49 | 1.67±1.63 | 0.182 |
| Frequency of stool 16^th^ 8 hours | 1.26±0.59 | 1.42±0.81 | 0.451 |
| Frequency of stool 17^th^ 8 hours | 1.59±1.37 | 1.67±0.82 | 0.238 |
| Frequency of stool 18^th^ 8 hours | 1.22±0.58 | 1.50±1.14 | 0.500 |
| Frequency of stool 19^th^ 8 hours | 1.28±0.46 | 1.36±0.68 | 0.902 |
| Frequency of stool 20^th^ 8 hours | 1.13±0.35 | 1.35±0.61 | 0.259 |
| Frequency of stool 21^st^ 8 hours | 1.11±0.32 | 1.30±0.70 | 0.355 |

Values are expressed as mean±SD; p-value was assessed by t-test or Chi-square test; SD, Standard deviation

**Table S5. Urine output per person in both the groups (ml)**

| **Variables (mean±SD)** | **Placebo (n=47)** | **Fibersol-2 (n=45)** | **p-value** |
| --- | --- | --- | --- |
| Urine output 1^st^ 8 hours | 113.83±44.44 | 118.20±44.18 | 0.637 |
| Urine output 2^nd^ 8 hours | 103.36±40.03 | 104.42±41.04 | 0.900 |
| Urine output 3^rd^ 8 hours | 101.55±36.53 | 103.98±37.70 | 0.756 |
| Urine output 4^th^ 8 hours | 114.68±40.37 | 110.48±37.60 | 0.639 |
| Urine output 5^th^ 8 hours | 117.87±34.76 | 108.14±40.95 | 0.224 |
| Urine output 6^th^ 8 hours | 104.78±29.14 | 99.00±32.61 | 0.378 |
| Urine output 7^th^ 8 hours | 117.02±36.41 | 115.24±45.62 | 0.837 |
| Urine output 8^th^ 8 hours | 117.66±37.92 | 114.67±42.16 | 0.721 |
| Urine output 9^th^ 8 hours | 104.53±30.64 | 118.02±38.44 | 0.065 |
| Urine output 10^th^ 8 hours | 113.91±39.69 | 116.67±40.11 | 0.743 |
| Urine output 11^th^ 8 hours | 123.40±43.70 | 115.56±33.27 | 0.337 |
| Urine output 12^th^ 8 hours | 113.21±38.46 | 112.22±37.59 | 0.901 |
| Urine output 13^th^ 8 hours | 124.48±36.54 | 123.67±42.51 | 0.987 |
| Urine output 14^th^ 8 hours | 128.91±53.31 | 124.11±41.82 | 0.633 |
| Urine output 15^th^ 8 hours | 122.21±39.08 | 103.43±30.46 | ­0.012 |
| Urine output 16^th^ 8 hours | 124.36±38.34 | 119.78±42.08 | 0.586 |
| Urine output 17^th^ 8 hours | 130.85±41.28 | 127.56±39.95 | 0.698 |
| Urine output 18^th^ 8 hours | 123.19±35.07 | 125.36±54.86 | 0.823 |
| Urine output 19^th^ 8 hours | 131.81±42.34 | 130.11±49.25 | 0.860 |
| Urine output 20^th^ 8 hours | 131.72±38.15 | 125.62±52.48 | 0.524 |
| Urine output 21^st^ 8 hours | 125.51±36.98 | 118.13±35.68 | 0.333 |

Values are expressed as mean±SD; p-value was assessed by t-test or Chi-square test; SD, Standard deviation

**Table S6. Amount of vomitus per day per person (gram)**

| **Variables (mean±SD)** | **Placebo (n=47)** | | **Fibersol-2 (n=45)** | | **p-value** |
| --- | --- | --- | --- | --- | --- |
|  |  | **n** |  | **n** |  |
| Amount of vomitus day 1 | 51.07±33.44 | 15 | 65.77±34.39 | 13 | 0.195 |
| Amount of vomitus day 2 | 85.63±60.09 | 8 | 78.33±48.56 | 3 | 1.000 |
| Amount of vomitus day 3 | 90.00±88.54 | 6 | 39.00±32.09 | 5 | 0.199 |
| Amount of vomitus day 4 | 75.00±73.99 | 5 | 72.67±25.33 | 3 | 0.549 |
| Amount of vomitus day 5 | 71.35±58.48 | 8 | 43.33±20.82 | 3 | 0.538 |
| Amount of vomitus day 6 | 20.00±17.32 | 3 | 30.00±0 | 1 | 0.637 |
| Amount of vomitus day 7 | 48.33±27.54 | 3 | 22.50±9.57 | 4 | 0.208 |

Values are expressed as mean±SD; p-value was assessed by t-test or Chi-square test; SD, Standard deviation

**Table S7. ORS intake per person in both groups (ml)**

| **Variables (mean±SD)** | **Placebo (n=47)** | **Fibersol-2 (n=45)** | **p-value** |
| --- | --- | --- | --- |
| ORS intake 1^st^ 8 hours | 337.17±340.32 | 308.49±307.00 | 0.813 |
| ORS intake 2^nd^ 8 hours | 226.34±156.90 | 270.24±183.68 | 0.202 |
| ORS intake 3^rd^ 8 hours | 242.56±255.14 | 288.65±336.40 | 0.287 |
| ORS intake 4^th^ 8 hours | 195.26±154.09 | 241.32±209.43 | 0.279 |
| ORS intake 5^th^ 8 hours | 204.39±181.97 | 236.45±168.00 | 0.188 |
| ORS intake 6^th^ 8 hours | 202.83±194.45 | 190.00±118.05 | 0.664 |
| ORS intake 7^th^ 8 hours | 195.00±173.56 | 187.92±138.15 | 0.927 |
| ORS intake 8^th^ 8 hours | 197.62±135.97 | 197.08±143.00 | 0.973 |
| ORS intake 9^th^ 8 hours | 145.00±121.99 | 241.58±179.39 | 0.064 |
| ORS intake 10^th^ 8 hours | 163.13±139.70 | 268.67±363.75 | 0.377 |
| ORS intake 11^th^ 8 hours | 175.56±142.84 | 198.75±249.96 | 0.555 |
| ORS intake 12^th^ 8 hours | 119.23±118.57 | 156.00±133.77 | 0.362 |
| ORS intake 13^th^ 8 hours | 212.00±162.47 | 217.27±205.87 | 0.887 |
| ORS intake 14^th^ 8 hours | 124.29±65.95 | 187.50±148.20 | 0.515 |
| ORS intake 15^th^ 8 hours | 96.36±60.54 | 162.00±136.94 | 0.301 |
| ORS intake 16^th^ 8 hours | 144.29±106.28 | 210.00±121.93 | 0.331 |
| ORS intake 17^th^ 8 hours | 135.00±84.56 | 160.00±96.18 | 0.711 |
| ORS intake 18^th^ 8 hours | 10.00±00 | 175.00±70.71 | 0.137 |
| ORS intake 19^th^ 8 hours | 170.00±75.83 | 147.50±73.20 | 0.620 |
| ORS intake 20^th^ 8 hours | 130.00±96.95 | 185.00±179.93 | 0.517 |
| ORS intake 21^st^ 8 hours | 100.00±00 | 185.00±88.88 | 0.480 |

Values are expressed as mean±SD; p-value was assessed by t-test or Chi-square test; SD, Standard deviation; ORS, Oral rehydration solution

**Table S8. Food intake per person (gram)**

| **Variables (mean±SD)** | **Placebo (n=47)** | | **Fibersol-2 (n=45)** | | **p-value** |
| --- | --- | --- | --- | --- | --- |
|  | **Food intake** | **n** | **Food intake** | **n** |  |
| Food intake 1^st^ 8 hours | 141.32±96.32 | 47 | 144.49±86.67 | 41 | 0.774 |
| Food intake 2^nd^ 8 hours | 131.22±56.36 | 46 | 128.20±62.67 | 45 | 0.841 |
| Food intake 3^rd^ 8 hours | 126.48±66.82 | 46 | 146.43±73.00 | 42 | 0.176 |
| Food intake 4^th^ 8 hours | 159.40±66.99 | 45 | 151.49±65.69 | 43 | 0.522 |
| Food intake 5^th^ 8 hours | 147.28±79.82 | 46 | 151.67±61.75 | 45 | 0.411 |
| Food intake 6^th^ 8 hours | 116.78±49.34 | 41 | 135.95±61.71 | 39 | 0.203 |
| Food intake 7^th^ 8 hours | 155.34±68.52 | 44 | 149.88±67.76 | 43 | 0.716 |
| Food intake 8^th^ 8 hours | 149.34±65.84 | 47 | 161.14±85.34 | 43 | 0.727 |
| Food intake 9^th^ 8 hours | 123.48±65.84 | 42 | 140.90±83.36 | 40 | 0.471 |
| Food intake 10^th^ 8 hours | 137.51±64.86 | 43 | 177.31±95.92 | 39 | 0.052 |
| Food intake 11^th^ 8 hours | 161.84±70.54 | 45 | 151.14±69.63 | 44 | 0.366 |
| Food intake 12^th^ 8 hours | 140.47±60.20 | 38 | 129.55±60.77 | 38 | 0.344 |
| Food intake 13^th^ 8 hours | 160.05±100.36 | 42 | 142.84±83.66 | 38 | 0.467 |
| Food intake 14^th^ 8 hours | 175.39±80.26 | 46 | 156.53±63.12 | 45 | 0.236 |
| Food intake 15^th^ 8 hours | 137.26±63.94 | 38 | 146.74±72.28 | 39 | 0.754 |
| Food intake 16^th^ 8 hours | 167.61±71.75 | 41 | 157.83±61.13 | 36 | 0.568 |
| Food intake 17^th^ 8 hours | 170.11±76.09 | 47 | 163.37±68.93 | 43 | 0.700 |
| Food intake 18^th^ 8 hours | 149.39±70.98 | 36 | 148.06±54.25 | 35 | 0.767 |
| Food intake 19^th^ 8 hours | 180.59±107.36 | 41 | 152.84±74.99 | 37 | 0.312 |
| Food intake 20^th^ 8 hours | 163.94±62.33 | 47 | 172.40±66.39 | 43 | 0.582 |
| Food intake 21^st^ 8 hours | 145.70±69.20 | 43 | 148.71±65.62 | 38 | 0.827 |

Values are expressed as mean±SD or number of subjects as n; p-value was assessed by t-test or Chi-square test; SD, Standard deviation

**Table S9. Formula milk intake per person (ml)**

| **Variables (mean±SD)** | **Placebo (n=47)** | | **Fibersol-2 (n=45)** | | **p- value** |
| --- | --- | --- | --- | --- | --- |
|  | **Formula milk intake** | **n** | **Formula milk intake** | **n** |  |
| Formula milk intake 1^st^ 8 hours | 153.33±128.58 | 3 | 96.67±89.63 | 3 | 0.275 |
| Formula milk intake 2^nd^ 8 hours | 220.00±243.31 | 3 | 85.00±20.21 | 2 | 0.767 |
| Formula milk intake 3^rd^ 8 hours | 300.00±141.41 | 2 | 60.00±0 | 1 | 0.221 |
| Formula milk intake 4^th^ 8 hours | 225.00±132.29 | 4 | 170.00±75.83 | 5 | 0.524 |
| Formula milk intake 5^th^ 8 hours | 250.00±100.00 | 3 | 100.00±0.00 | 4 | 0.019 |
| Formula milk intake 6^th^ 8 hours | 192.50±50.58 | 4 | 85.00±21.21 | 2 | 0.060 |
| Formula milk intake 7^th^ 8 hours | 167.50±66.52 | 4 | 125.00±35.35 | 2 | 0.481 |
| Formula milk intake 8^th^ 8 hours | 158.00±67.60 | 5 | 100.00±0.00 | 2 | 0.421 |
| Formula milk intake 9^th^ 8 hours | 120.00±42.43 | 2 | 150.00±70.71 | 2 | 0.658 |
| Formula milk intake 10^th^ 8 hours | 165.00±120.21 | 2 | 0 | 0 | - |
| Formula milk intake 11^th^ 8 hours | 117.50±23.63 | 4 | 106.67±66.58 | 3 | 0.476 |
| Formula milk intake 12^th^ 8 hours | 150.00±91.29 | 4 | 125.00±0 | 1 | 1.000 |
| Formula milk intake 13^th^ 8 hours | 130.00±24.50 | 4 | 120±0 | 1 | 0.709 |
| Formula milk intake 14^th^ 8 hours | 135.00±55.08 | 4 | 115.00±49.50 | 2 | 0.481 |
| Formula milk intake 15^th^ 8 hours | 225.00±35.36 | 2 | 90.00±42.43 | 2 | 0.121 |
| Formula milk intake 16^th^ 8 hours | 140.00±54.77 | 5 | 125.00±0 | 1 | 0.752 |
| Formula milk intake 17^th^ 8 hours | 143.33±92.38 | 3 | 130.00±0 | 1 | 0.637 |
| Formula milk intake 18^th^ 8 hours | 150.00±0 | 1 | 160.00±60.00 | 3 | 0.655 |
| Formula milk intake 19^th^ 8 hours | 143.75±71.81 | 4 | 175.00±106.07 | 2 | 0.803 |
| Formula milk intake 20^th^ 8 hours | 146.00±55.50 | 5 | 150.00±100.00 | 3 | 1.000 |
| Formula milk intake 21^st^ 8 hours | 129.00±41.29 | 5 | 123.33±25.17 | 3 | 1.000 |

Values are expressed as mean±SD or number of subjects as n; p-value was assessed by t-test or Chi-square test; SD, Standard deviation
